# Supplementary material for: Mask Wearing and Control of SARS-CoV-2 Transmission in the United States
Source: medRxiv. 2020 Sep 1:2020.08.23.20078964. Preprint. [Version 3] doi: 10.1101/2020.08.23.20078964 (PMC7457618; doi:10.1101/2020.08.23.20078964)
Supplement: 1 [file NIHPP2020.08.23.20078964-supplement-1.pdf]

medRxiv preprint doi: https://doi.org/10.1101/2020.08.23.20078964.this version posted September 1, 2020. The copyright holder for this preprint (which was not certified by peer review) is the author/funder, who has granted medRxiv a license to display the preprint in perpetuity.
It is made available under a CC-BY 4.0 International license .

Supplementary Materials

Figure S1. Mask Wearing, Social Contacts and the Predicted Probability of *Rt* below 1

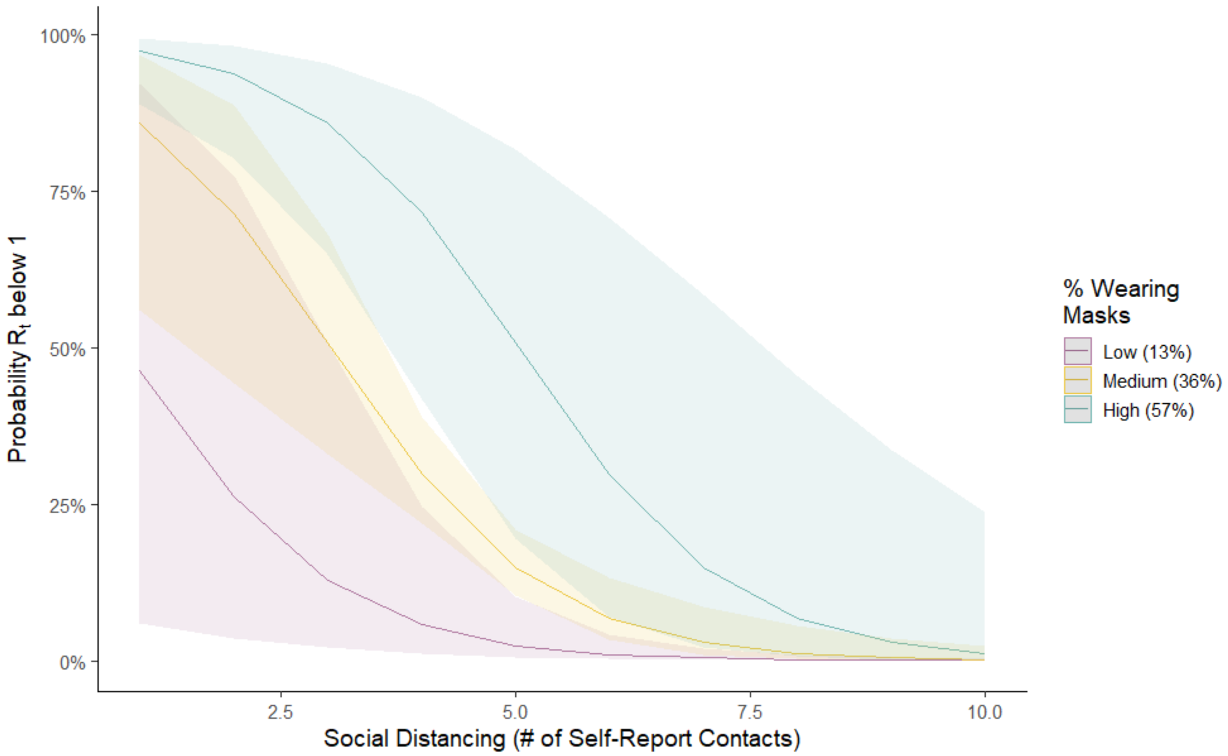

Projected values from a logistic regression model measuring the association of community transmission control (R_t <1) with mask-wearing and social contacts in US states adjusting for population density, percent non-white and a time trend (Model 1). The number of self-reported contacts at “social gatherings” from Facebooks’ COVID-19 symptom survey was aggregated over each week and state utilizing a weighted sampling scheme.

medRxiv preprint doi: https://doi.org/10.1101/2020.08.23.20078964.this version posted September 1, 2020. The copyright holder for this preprint
(which was not certified by peer review) is the author/funder, who has granted medRxiv a license to display the preprint in perpetuity.
It is made available under a CC-BY 4.0 International license .

Figure S2. Association of mask wearing with R_t at different dichotomization cutoffs

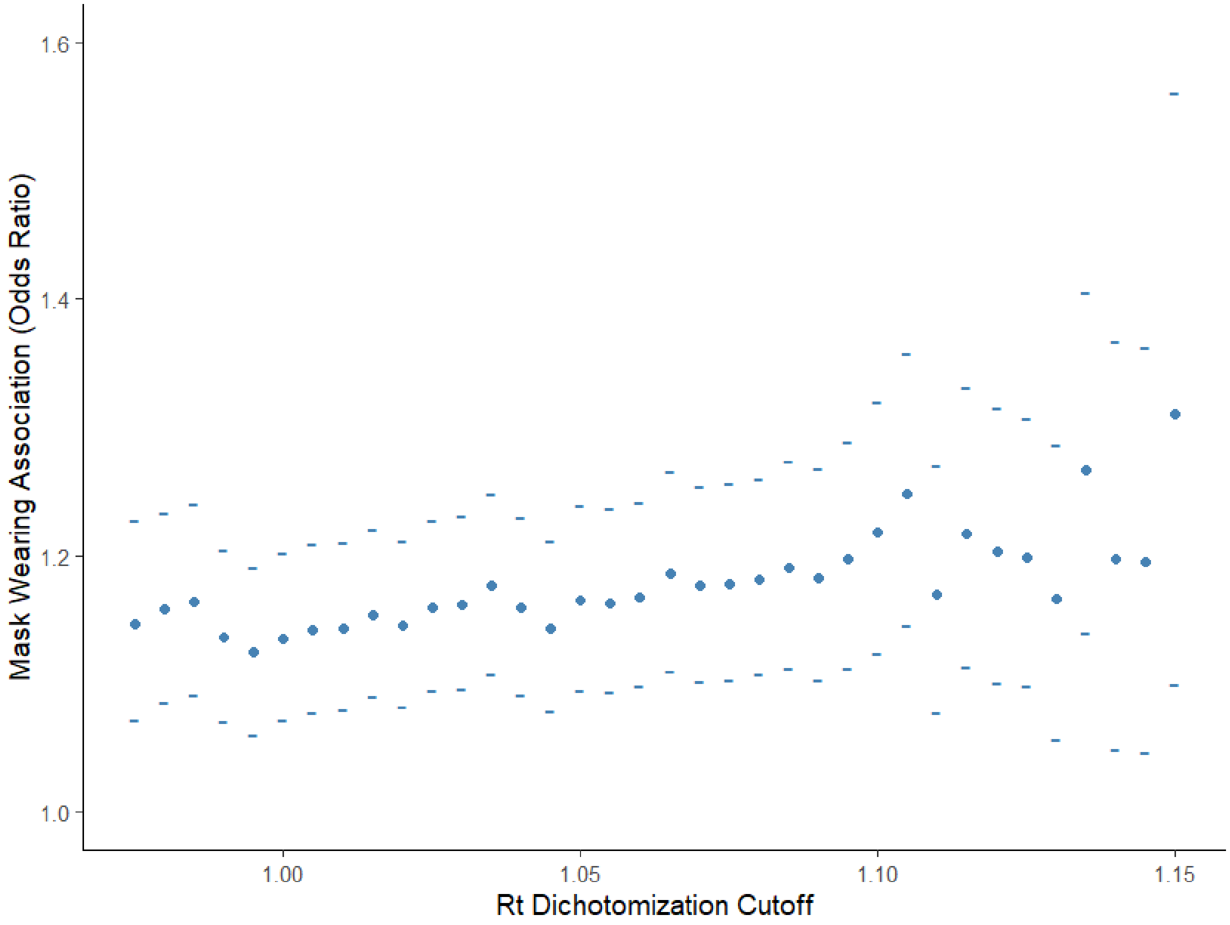

Results from a logistic regression model measuring the association of community transmission control ($R_t < x$) with mask-wearing adjusting for social distancing, population density, percent non-white and a time trend. Model was repeated as cutoff for R_t dichotomization (x) was varied. The odds ratio (point) and 95% confidence interval (-) for mask wearing that resulted from each iteration is shown.

medRxiv preprint doi: https://doi.org/10.1101/2020.08.23.20078964.this version posted September 1, 2020. The copyright holder for this preprint
(which was not certified by peer review) is the author/funder, who has granted medRxiv a license to display the preprint in perpetuity.
It is made available under a CC-BY 4.0 International license .

Figure S3. Association of mask wearing with categorical R_t

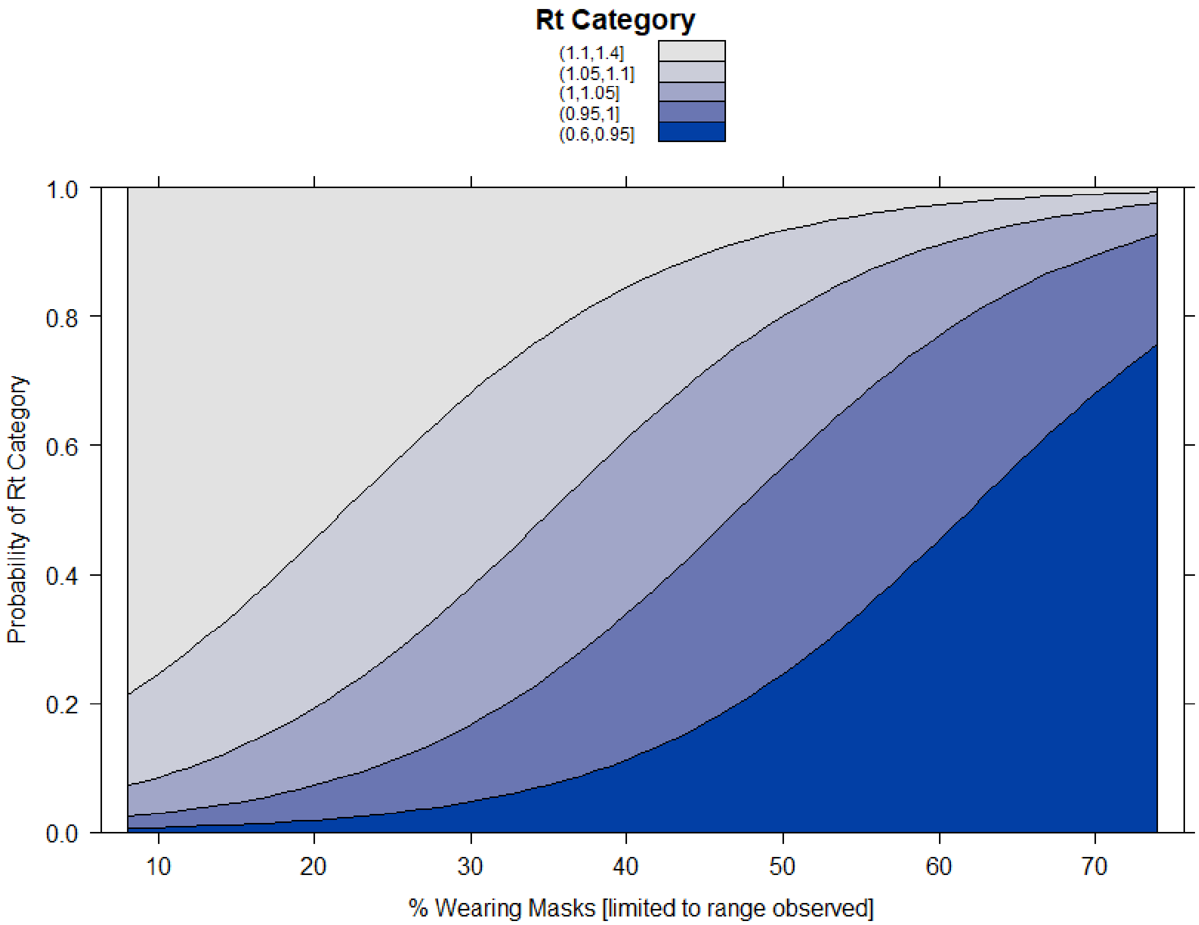

Projected probabilities from an ordinal logistic regression model measuring the association of community transmission control (R_t) with mask-wearing adjusting for social distancing, population density, percent non-white and a time trend. Observed mask wearing was between 8.1%-73.7%.
